# Translation, cultural adaptation, and validation of the amharic version of the Minnesota Living with Heart Failure Questionnaire (MLHFQ)

**DOI:** 10.1186/s41687-026-01089-y

**Published:** 2026-05-26

**Authors:** Henok Mulugeta, Peter M. Sinclair, Suzanne Sheppard-Law, Amanda Wilson

**Affiliations:** 1 Department of Nursing, College of Health Sciences, Debre Markos University, Debre Markos, Amhara Region Ethiopia; 2 School of Nursing and Midwifery, Faculty of Health, University of Technology Sydney, Sydney, New South Wales Australia

**Keywords:** Heart failure, MLHFQ, HRQoL, Psychometric properties, Reliability, Validity

## Abstract

**Background:**

Heart failure has a significant impact on the health-related quality of life of affected individuals. The Minnesota Living with Heart Failure Questionnaire (MLHFQ) is one of the most widely used instruments for measuring the health-related quality of life of people with heart failure. This study aimed to translate, culturally adapt, and validate the psychometric properties of the Amharic MLHFQ among people with heart failure in Ethiopia.

**Methods:**

A hospital-based cross-sectional study was conducted among adults with heart failure attending the cardiac outpatient clinics of two tertiary-level public hospitals in Ethiopia. The health-related quality of life of these people was measured using the Amharic version of the MLHFQ. The study was conducted in two phases: translation and cultural validation, followed by psychometric testing to assess the reliability and validity of the instrument. The translation and cross-cultural adaptation process involved forward translation, synthesis, back translation, expert panel review, and pre-testing. The scale level content validity index (S-CVI) was calculated to assess content validity. The internal consistency of the tool was evaluated using reliability test, while construct validity was examined using confirmatory factor analysis (CFA).

**Results:**

A total of 383 adults with heart failure participated in the study. The scale-level content validity index (S-CVI) was 0.87, indicating good content validity. The Cronbach’s α coefficient for the Amharic version of the MLHFQ was 0.90, demonstrating strong internal consistency. Confirmatory factor analysis (CFA) results revealed that the two-factor model provided an acceptable fit to the data (CFI = 0.92, RMSEA = 0.10, SRMR = 0.10, χ^2^ = 278.047, *p* < 0.001). There were significant differences in MLHFQ scores across age groups, depression status, New York Heart Association (NYHA) class and social support levels.

**Conclusion:**

The Amharic version of the MLHFQ demonstrated good reliability and acceptable validity, supporting its use as a culturally appropriate tool for assessing the health-related quality of life of Amharic-speaking Ethiopian people with heart failure. Health care providers can use this tool in both clinical practice and research settings to better evaluate and monitor patient-reported outcomes. Future research is recommended to confirm and strengthen the findings of this study.

## Background

Heart failure (HF) is a major cardiovascular disease affecting more than 64 million people [1], and the primary cause of over 300,000 annual deaths globally [2]. In Ethiopia, the prevalence and burden of HF is increasing particularly among those aged 15–39 years [3]. It has significant adverse outcomes, including prolonged hospital stay, poor health-related quality of life (HRQoL), and high mortality rates [4, 5].

Heart failure is a chronic condition associated with impairment of cardiac function that affects physical, mental, and social health [6, 7]. It has a significant impact on the health care system due to high readmission rates and associated costs [1]. People with HF have poorer HRQoL compared to the normal population due to the physical, emotional, and social disruption throughout the progression of the disease [8]. Poor HRQoL has been associated with a poorer prognosis, which, in turn, is a significant predictor of rehospitalisation [9, 10].

Health-related quality of life (HRQoL) is an important measurement of overall well-being and health status, especially in people with chronic conditions like HF, where the impact on various aspects of life is significant [11, 12]. It is a multidimensional concept consisting of physical, social, emotional, psychological, and mental functioning [13, 14]. The Minnesota Living with Heart Failure Questionnaire (MLHFQ) is the most widely used disease-specific instrument for assessing HRQoL in people with HF. [15, 16]. It is a 21-item questionnaire with a 6-point Likert-type scale that ranges from 0 (no effect) to 5 (very much effect). The original questionnaire was developed in the USA in 1986 [17]. The scores range from 0 to 105, with higher composite scores representing a poorer quality of life [18–20]. The MLHFQ provides a total score as well as scores on two dimensions: physical (eight questions, range 0–40) and emotional (five questions, range 0–25) [19, 20].

The MLHFQ has been translated and validated in different languages and cultures, including the United Kingdom (UK) [21], Spain [22, 23], the Netherlands [24], Taiwan [25], Korea [26], Brazil [27], China [28], and Lebanon [29]. However, it has not yet been translated, culturally adapted, or psychometrically validated for use in Ethiopia in the Amharic language. This study aimed to translate, culturally adapt and validate the psychometric properties of the Amharic version of the MLHFQ among people living with HF in Ethiopia. Given the significant impact of HF on various aspects of life [30] and the high prevalence of poor HRQoL among Ethiopians with HF [31], there is a need for valid and reliable tool to help clinicians in assessing HRQoL in this population. A validated Amharic version of MLHFQ would also contribute to future research on HRQoL of people with HF across different settings in Ethiopia, providing accurate and reliable data for both clinical and research purposes.

## Methods

### Study design, setting and period

This psychometric study was based on data from a hospital-based cross-sectional study conducted to assess the health-related quality of life of people living with heart failure in Ethiopia. The study was conducted between 21 November 2022 and 22 January 2023 at the cardiac outpatient clinics of two government hospitals in Addis Ababa, the capital city of Ethiopia: St. Paul’s Hospital Millennium Medical College and St. Peter Specialised Hospital. These are among the largest tertiary-level hospitals in the country and provide cardiology services and treatment for patients from all around the country.

### Study population and eligibility

The inclusion criteria for this study were adults aged 18 years and above, diagnosed with HF, and undergoing a minimum of three months of follow-up care at the outpatient cardiac clinic at either of the two hospitals. Anyone unable or unwilling to provide informed consent was excluded.

### Sample size determination and recruitment

A total sample size of 383 was calculated using the single population proportion formula (*N*=(Zα/2)^2^ *P(1 − P)2/D^2^) [32] with the assumption of a 95% confidence interval, marginal error (d) of 5.0% and 51.8% prevalence (P) of low HRQoL [33]. Participants were recruited using a consecutive sampling technique, whereby eligible individuals were enrolled sequentially based on their availability and accessibility until the required sample size was reached.

### Data collection and study procedures

Data were collected using an interviewer-administered structured questionnaire consisting of four sections. The first section assessed sociodemographic and clinical related characteristics; the second dealt with social support, the third measured depression, and the final section evaluated HRQoL. People with HF who met the eligibility criteria were approached by trained research assistants (RAs) during their routine follow-up visits at the outpatient cardiac clinics of each hospital. Data were collected in person using an interviewer-administered questionnaire.

Sociodemographic and clinical characteristics were assessed using a 19-item questionnaire that gathered information on age, sex, marital status, employment status, residence, educational level, health insurance, family history of HF, hospitalisation history, comorbidities, duration of illness, NYHA class, number of medications taken daily and general health perception. The Oslo Social Support Scale (OSSS-3) was used to measure the level of social support, while depression was measured using the patient health questionnaire (PHQ-9). Finally, the Minnesota Living with Heart Failure Questionnaire (MLHFQ) was used to measure the health-related quality of life of participants.

This study involved two phases: translation and cultural adaptation, and psychometric testing. The translation and cross-cultural adaptation process was undertaken following the guidelines proposed by Beaton et al. (2000) and involved the following steps: forward translation, synthesis, back translation, expert committee review, and pre-testing [34]. Permission to translate and adapt the original MLHFQ into Amharic was obtained from the University of Minnesota, the copyright holder. The license granted allows for research use of the adapted version within Ethiopia. Any further modification or distribution beyond research purposes would require additional permission from the copyright holder. The psychometric tests performed included, a reliability test (internal consistency), and validation (face validity, content validity, construct validity). The internal consistency reliability of the Amharic MLHFQ was assessed using Cronbach’s alpha, inter-item correlations, and split-half reliability, while construct validity was evaluated using confirmatory factor analysis (CFA) and by examining convergent, divergent, and known-groups validity.

### Data analysis

Collected data were cleaned and entered into Epi-Data version 3.1 and exported to STATA Version 17 for analysis [35]. Descriptive statistics were used to summarise the socio-demographic and clinical characteristics of the participants, as well as the OSSS-3, PHQ-9, and MLHFQ scores. The Cronbach’s alpha (α) coefficient was calculated to evaluate the internal-consistency reliability of the MLHFQ. Face and content validity of the translated instrument were evaluated by a panel of experts in the field. Confirmatory factor analysis (CFA) was performed to assess the goodness-of-fit of the two-factor structure of the MLHFQ as identified in the original study [36]. Model fit was evaluated using absolute and comparative fit indices, including the chi-square goodness-of-fit (χ^2^), the Non-normed Fit Index (NFI), the Root Mean Square Error of Approximation (RMSEA), Standardized Root Mean Square Residual (SRMR) and the Comparative Fit Index (CFI). The CFA was performed using AMOS version 22 (IBM SPSS). Convergent and divergent validity were assessed using Pearson’s correlation coefficients between the Amharic MLHFQ scores and other measures (PHQ-9 and OSSS). Group comparisons of MLHFQ scores across several demographic and clinical variables were evaluated using known group validity. It was hypothesized that patients with worse sociodemographic and clinical status would have higher MLHFQ scores. Group differences were examined using t-test or ANOVA, and appropriate effect sizes (Cohen’s d or η^2^) were calculated. A *p*-value of <0.05 was considered statistically significant.

## Results

### Sociodemographic characteristics of the study participants

A total of 383 adults with HF participated in this study with a response rate of 100%. Of these, 184 (48.04%) were male and 199 (51.96%) were female with a mean age of 55.1 ± 15.38 years. A total of 196 (51.17%) participants were married, and 184 (48.04%) were employed. Most, 286 (74.67%), lived in urban areas, and 114 (29.77%) had low or no formal education (unable to read and write). The majority, 276 (72.06%), of the study participants were treated under the community health insurance (CHI) scheme. Details of the socio-demographic characteristics of the participants are summarised in Table 1.

**Table 1:** Sociodemographic characteristics of participants (*n* = 383), 2023

Variables	Category	Frequency (%)
Age in years	18–39 40–69 ≥70	74 (19.32) 222 (57.96) 87 (22.72)
Sex	Male Female	184 (48.04) 199 (51.96)
Marital status	Single Married Divorced Widowed Separated	65 (16.97) 196 (51.17) 33 (8.62) 69 (18.02) 20 (5.22)
Employment status	Employed Unemployed	184 (48.04) 199 (51.96)
Residence	Urban Rural	286 (74.67) 97 (25.33)
Educational level	Low or no education Primary education Secondary education College and above	114 (29.77) 108 (28.20) 87 (22.72) 74 (19.32)
Community health insurance	Yes No	276 (72.06) 107 (27.94)

### Clinical characteristics of the study participants

Most participants (87.73) had no family history of HF, and 132 (34.46%) had a history of hospitalisation in the previous twelve months. The mean duration of HF diagnosis was 2.5 years, and 276 of participants (72.06%) were taking fewer than five medications daily. Additionally, 169 (44.13%) and 80 (20.89%) participants had comorbid hypertension and diabetes, respectively and 135 (35.25%) were classified in NYHA class I. The mean social support score (OSSS-3) was 8.98 ± 2.94, the mean depression score (PHQ-9) was 11.02 ± 6.14, and thee mean total MLHFQ score was 48.03 ± 19.73 (Table 2).

**Table 2:** Clinical characteristics of the participants (*n* = 383), 2023

Variables	Category	Frequency (%)
Family history of heart failure	No Yes	336 (87.73) 47 (12.27)
Hospitalization in the past 12 months	No Yes	251 (65.54) 132 (34.46)
Comorbidities		
Hypertension	Yes	169 (44.13)
Diabetes	Yes	80 (20.89)
Kidney disease	Yes	29 (7.57)
COPD and asthma	Yes	11 (2.87)
Cancer	Yes	3 (0.78)
HIV/AIDS	Yes	19 (4.96)
Duration of HF diagnosis	<1 year 1–5 years 5–10 years 10–15 years >15 years	65 (16.97) 154 (40.21) 107 (27.94) 34 (8.88) 23 (6.01)
Medications taken daily	<5 ≥5	276 (72.06) 107 (27.94)
NYHA class	Class I Class II Class III Class IV	135 (35.25) 117 (30.55) 93 (24.28) 38 (9.92)
General health perception	Excellent Very good Fair Poor	32 (8.36) 102 (26.63) 149 (38.90) 100 (26.11)
PHQ-9 score	Mean score	11.02 ± 6.14
OSSS-3 score	Mean ± SD	8.98 ± 2.94
MLHFQ score	Mean ± SD	48.03 ± 19.73

### Item level descriptive statistics and response distribution

The item-level analysis showed a generally balanced distribution of responses across categories. The item means ranged from 1.54 to 3.24, and the Skewness and kurtosis values were within acceptable ranges, supporting the assumption of normality. Detailed item characteristics, including frequencies for each response category, item means and standard deviations, and distributional properties (skewness and kurtosis), are presented in Table 3.

**Table 3:** Item-level descriptive statistics and response distribution (*n* = 383), 2023

No	Item	Response n (%)						Mean (SD)	Skewness	Kurtosis
		0	1	2	3	4	5			
01	causing swelling in your ankles or legs?	124(32.4)	97(25.3	45(11.7)	38(9.9)	54(14.1)	25(6.5)	1.68 ± 1.64	0.64	−0.93
02	making you sit or lie down to rest during the day	28(7.3)	105(27.4)	92(24.0)	96(25.1)	49(12.8)	13(3.4)	2.19 ± 1.27	0.21	−0.71
03	making your walking about or climbing stairs difficult?	14(3.7)	45(11.7)	53(13.8)	78(20.4)	109(28.5)	84(21.9)	3.24 ± 1.43	−0.52	−0.71
04	making your working around the house or yard difficult?	35(9.1)	79(20.6)	52(13.6)	104(27.2)	84(21.9)	29(7.6)	2.55 ± 1.45	−0.16	−1.01
05	making your going places away from home difficult?	32(8.4)	38(9.9)	46(12.0)	74(19.3)	111(29.0)	82(21.4)	3.15 ± 1.55	−0.61	−0.69
06	making your sleeping well at night difficult?	90(23.5)	57(14.9)	64(16.7)	65(17.0)	62(16.2)	45(11.7)	2.23 ± 1.71	0.12	−1.26
07	making your relating to or doing things with your friends or family difficult?	111(29.0)	105(27.4)	76(19.8)	41(10.7)	39(10.2)	11(2.9)	1.54 ± 1.42	0.70	−0.49
08	making your working to earn a living difficult?	58(15.1)	40(10.4)	29(7.6)	76(19.8)	90(23.5)	90(23.5)	2.97 ± 1.74	−0.49	−1.08
09	making your recreational pastimes, sports or hobbies difficult?	78(20.4)	111(29.0)	42(11.0)	59(15.4)	57(14.9)	36(9.4)	2.04 ± 1.65	0.38	−1.15
10	making your sexual activities difficult?	172(44.9)	50(13.1)	26(6.8)	41(10.7)	64(16.7)	30(7.8)	1.65 ± 1.81	0.57	−1.27
11	making you eat less of the foods you like?	75(19.6)	61(15.9)	84(21.9)	82(21.4)	57(14.9)	24(6.3)	2.15 ± 1.52	0.10	−1.01
12	making you short of breath?	27(7.0)	47(12.3)	53(13.8)	105(27.4)	91(23.8)	60(15.7)	2.96 ± 1.46	−0.41	−0.71
13	making you tired, fatigued, or low on energy?	16(4.2)	45(11.7)	51(13.3)	83(21.7)	119(31.1)	69(18.0)	3.18 ± 1.41	−0.54	−0.62
14	making you stay in a hospital?	80(20.9)	50(13.1)	85(22.2)	89(23.2)	55(14.4)	24(6.3)	2.16 ± 1.52	0.05	−0.01
15	costing you money for medical care?	46(12.0)	86(22.5)	82(21.4)	69(18.0)	69(18.0)	31(8.1)	2.32 ± 1.50	0.14	−1.03
16	giving you side effects from treatments?	82(21.4)	96(25.1)	73(19.1)	60(15.7)	43(11.2)	29(7.6)	1.93 ± 1.55	0.45	−0.87
17	making you feel you are a burden to your family or friends	106(27.7)	82(21.4)	61(15.9)	65(17.0)	37(9.7)	32(8.4)	1.85 ± 1.62	0.47	−0.94
18	making you feel a loss of self-control in your life?	127(33.2)	85(22.2)	69(18.0)	56(14.6)	34(8.9)	12(3.1)	1.53 ± 1.46	0.62	−0.67
19	making you worry?	72(18.8)	74(19.3)	49(12.8)	71(18.5)	63(16.4)	54(14.1)	2.37 ± 1.71	0.07	−1.30
20	making it difficult for you to concentrate or remember things?	76(19.8)	67(17.5)	53(13.8)	68(17.8)	60(15.7)	59(15.4)	2.38 ± 1.74	0.06	−1.31
21	making you feel depressed?	76(19.8)	90(23.5)	74(19.3)	67(17.5)	56(14.6)	20(5.2)	1.99 ± 1.51	0.29	−1.01

### Translation

The standard forward–backwards procedure was applied to translate the MLHFQ from English into the local Ethiopian language, Amharic [37]. Two independent translators fluent in both English and Amharic and familiar with Ethiopian culture, conducted the initial translation from the English to the local language, Amharic. The research team and translators met to synthesise the results of the translations and resolve any discrepancies. The initial translation was then independently back translated from the synthesised Amharic version into the original English language to ensure the accuracy of the translation. The original and back-translated versions were reviewed by a panel of experts, including researchers, translators, and health professionals in the field, to develop the prefinal version for pre-testing. Finally, the prefinal version was tested on a sample of 40 people with HF to check the understanding, interpretation, and cultural relevance of the translated MLHFQ. Participants were recruited using a consecutive sampling technique, and the questionnaire was administered in a face-to-face setting. Participants were asked to provide feedback on item clarity, wording and any difficulties in understanding the questions. The pretest results showed that most items were clear to understand and culturally acceptable. However, minor wording issues were identified in some items, and the necessary corrections were made to improve clarity. No major modifications to the questionnaire structure were required. The preliminary pre-testing helped identify and address any issues with question comprehension or cultural applicability.

### Cultural adaptation

A panel of six experts, each a subject matter expert, researcher, and professional in the field (specialised in cardiology and mental health), evaluated the translated MLHFQ for its suitability within the cultural and linguistic context of Ethiopia. Cultural adaptation involves considering the socio-cultural context of the target population in relation to the questionnaire. The experts assessed the clarity, appropriateness, and unambiguousness of the items in the tool in relation to Ethiopian culture. All experts agreed that all items were culturally appropriate and relevant to the Ethiopian context. However, experts suggested changing the wording of a few questions to allow a better understanding of the items for the respondents. Necessary corrections of wording were made using locally spoken and culturally acceptable words. The final culturally adapted Amharic version of the MLHFQ, along with the original English version, is presented in Table 4.

**Table 4:**
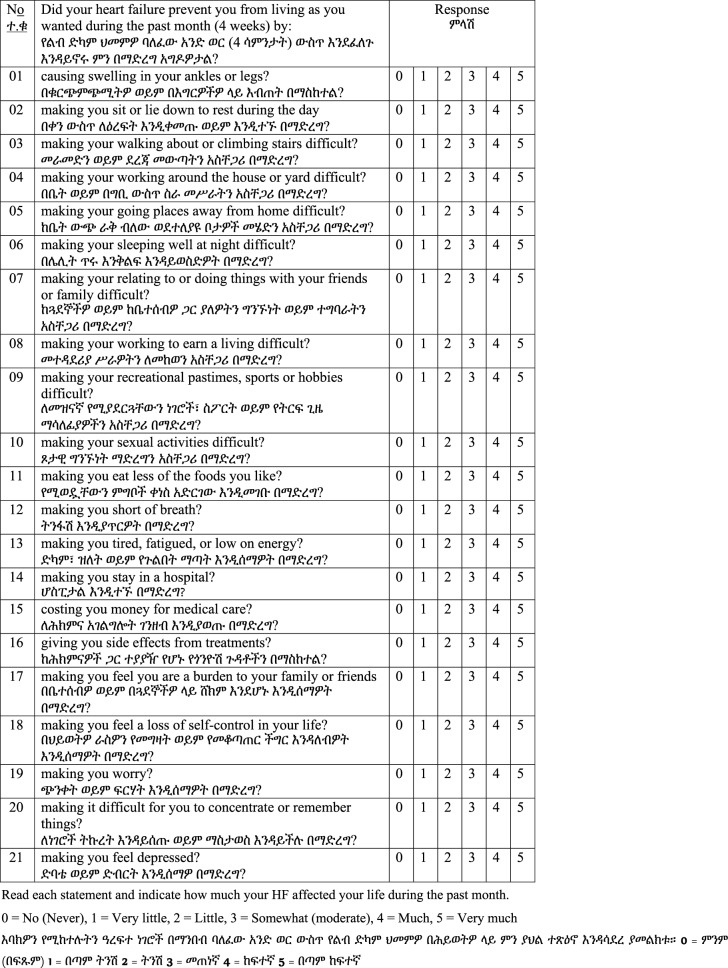
Culturally adapted Amharic MLHFQ alongside the original English version, 2023

### Reliability (Internal consistency)

Cronbach’s alpha (α) coefficients were calculated to evaluate the internal consistency of the MLHFQ. Cronbach’s alpha (α) coefficients range from 0 to 1, with higher values indicating that items are more strongly interrelated with one another [38]. The analysis showed that the Cronbach’s α coefficient of the total MLHFQ was 0.90 and split-half reliability of 0.94, indicating excellent internal consistency. The physical and emotional subscales demonstrated good internal consistency, with Cronbach’s alpha of 0.87 (split-half coefficients = 0.89) and 0.84 (split-half coefficients = 0.86), respectively (Table 5).

**Table 5:** Reliability of the Amharic MLHFQ scores (*n* = 383), 2023

MLHFQ	Cronbach’s alpha (α)	Split-half reliability (Spearman–Brown)
Total scale	0.90	0.94
Physical subscale	0.87	0.89
Emotional subscale	0.84	0.86

In addition, there were no inter-item correlations with values greater than 0.80 (Table 6). An inter-item correlation between 0.2 and 0.8 is considered acceptable for items measuring the same construct [39, 40].

**Table 6:** Inter-item correlation matrix of the Amharic MLHFQ (*n* = 383), 2023

	Q1	Q2	Q3	Q4	Q5	Q6	Q7	Q8	Q9	Q10	Q11	Q12	Q13	Q14	Q15	Q16	Q17	Q18	Q19	Q20	Q21
Q1	1																				
Q2	0.48	1																			
Q3	0.25	0.43	1																		
Q4	0.28	0.37	0.64	1																	
Q5	0.29	0.38	0.73	0.76	1																
Q6	0.24	0.23	0.38	0.49	0.4	1															
Q7	0.23	0.22	0.28	0.38	0.32	0.35	1														
Q8	0.25	0.36	0.5	0.55	0.48	0.33	0.44	1													
Q9	0.26	0.23	0.29	0.37	0.27	0.32	0.54	0.56	1												
Q10	0.3	0.28	0.27	0.22	0.26	0.23	0.25	0.32	0.22	1											
Q11	0.21	0.36	0.4	0.41	0.36	0.54	0.39	0.45	0.34	0.31	1										
Q12	0.27	0.32	0.67	0.55	0.55	0.44	0.33	0.47	0.41	0.22	0.5	1									
Q13	0.21	0.28	0.65	0.55	0.56	0.39	0.33	0.47	0.41	0.27	0.42	0.73	1								
Q14	0.23	0.27	0.32	0.4	0.35	0.26	0.4	0.25	0.41	0.08	0.3	0.34	0.37	1							
Q15	0.27	0.27	0.25	0.27	0.25	0.21	0.27	0.28	0.35	0.27	0.29	0.21	0.23	0.48	1						
Q16	0.28	0.24	0.34	0.33	0.26	0.35	0.41	0.22	0.45	0.23	0.35	0.37	0.35	0.51	0.43	1					
Q17	0.28	0.29	0.22	0.34	0.28	0.28	0.43	0.4	0.46	0.09	0.25	0.24	0.29	0.34	0.42	0.45	1				
Q18	0.26	0.31	0.28	0.42	0.36	0.22	0.54	0.49	0.46	0.23	0.4	0.34	0.28	0.35	0.34	0.45	0.62	1			
Q19	0.22	0.28	0.37	0.38	0.4	0.41	0.34	0.31	0.45	0.23	0.28	0.42	0.4	0.41	0.24	0.42	0.43	0.47	1		
Q20	0.22	0.23	0.44	0.45	0.42	0.46	0.35	0.35	0.43	0.25	0.38	0.54	0.5	0.39	0.09	0.47	0.3	0.42	0.68	1	
Q21	0.28	0.26	0.28	0.31	0.27	0.27	0.5	0.37	0.48	0.28	0.36	0.28	0.29	0.43	0.32	0.4	0.5	0.58	0.58	0.54	1

### Validation: face validity, content validity, construct validity 

#### Face and content validity

Face validity, the subjective judgment of the tool, was evaluated in terms of clarity, relevance, and appropriateness of the items. To assess the content validity of the tool, a Content Validity Index (CVI) was used, with evaluations provided by a panel of six experts. The CVI quantifies the degree to which experts (E) agree that the items in the tool are relevant and representative of the construct being measured [41]. Both the item-level CVI (I-CVI) and scale-level CVI (S-CVI) were calculated. For each item, the I-CVI represents the number of experts who rated the item as 3 or 4 (indicating relevance) divided by the total number of experts. The S-CVI is the average of all I-CVI values across the scale, providing an overall measure of the content validity of the scale. The analysis revealed that the overall S-CVI was 0.87. An S-CVI of 0.83 or higher is generally considered acceptable for the overall scale, consistent with methodological recommendations for a panel of six or more experts [42, 43]. The detailed results of the CVI analysis are summarized in Table 7.

**Table 7:** Content validity index (CVI) analysis of the Amharic MLHFQ, 2023

No	Did your heart failure prevent you from living as you wanted during the past month by: -	E1	E2	E3	E4	E5	E6	I-CVI
01	causing swelling in your ankles or legs?	4	3	3	4	4	4	1.00
02	making you sit or lie down to rest during the day	3	3	3	4	4	4	1.00
03	making your walking about or climbing stairs difficult?	4	3	4	3	4	3	1.00
04	making your working around the house or yard difficult?	3	2	3	3	3	3	0.83
05	making your going places away from home difficult?	4	3	4	4	4	4	1.00
06	making your sleeping well at night difficult?	3	3	3	4	3	4	1.00
07	making your relating to or doing things with your friends or family difficult?	3	3	2	3	2	3	0.67
08	making your working to earn a living difficult?	3	3	2	4	4	3	0.83
09	making your recreational pastimes, sports or hobbies difficult?	4	2	2	2	3	3	0.50
10	making your sexual activities difficult?	3	3	3	2	2	3	0.67
11	making you eat less of the foods you like?	4	3	3	3	4	3	1.00
12	making you short of breath?	4	3	3	4	3	4	1.00
13	making you tired, fatigued, or low on energy?	4	4	4	4	4	4	1.00
14	making you stay in a hospital?	3	2	3	3	2	3	0.67
15	costing you money for medical care?	4	3	3	3	4	3	1.00
16	giving you side effects from treatments?	3	3	3	3	3	3	1.00
17	making you feel you are a burden to your family or friends	3	4	3	3	3	4	1.00
18	making you feel a loss of self-control in your life?	3	3	2	2	2	3	0.50
19	making you worry?	4	3	3	3	4	4	1.00
20	making it difficult for you to concentrate or remember things?	3	3	3	2	2	3	0.67
21	making you feel depressed?	3	4	3	3	4	4	1.00
	Scale-level CVI (S-CVI)							0.87

### Construct validity

**Fig. 1:**
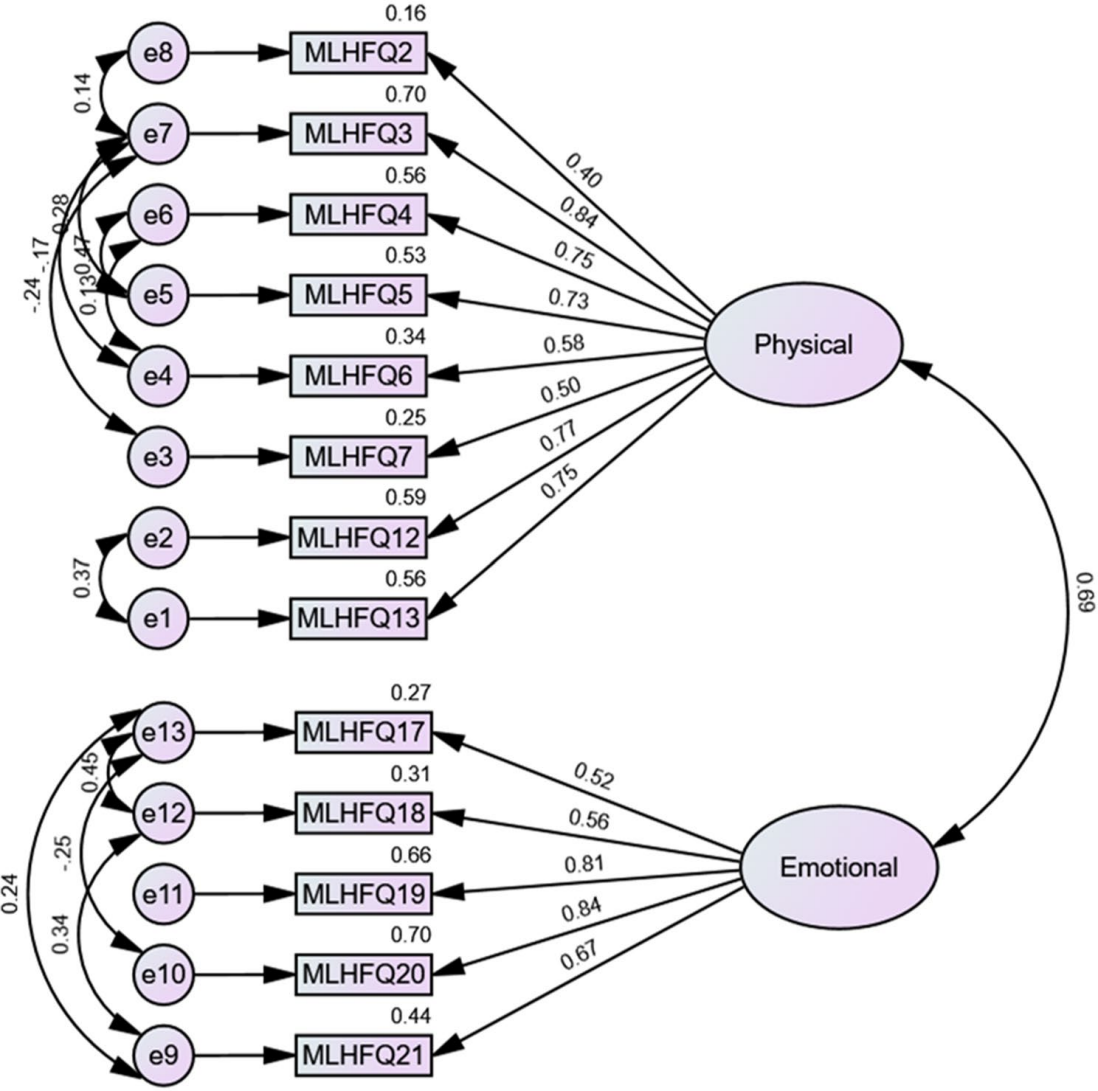
Confirmatory factor analysis (CFA) model of the Amharic MLHFQ (*n* = 383)

The result of the CFA for the MLHFQ two-factor model showed an acceptable but marginal overall fit of the data to the model (CFI = 0.92, RMSEA = 0.10, SRMR = 0.10, x2 = 278.047 with *p* < 0.001). The loadings of items on the corresponding factors were generally good and positive, ranging from 0.50 to 0.84 for all items except for item 2, which was borderline at 0.40 (Fig. 1). Loadings greater than ±0.33 are considered to meet the minimal level of practical significance [39, 44]. A correlation analysis, as part of the construct validity of the MLHFQ, was also conducted to test its hypothesised correlation with other relevant variables.

### Convergent and divergent validity

The convergent and divergent validity of the Amharic MLHFQ was evaluated by its correlation with PHQ-9 and OSS. The Pearson correlation analysis showed that the Amharic MLHFQ and its subscales strongly correlated with PHQ-9 scores, supporting convergent validity. In contrast, Amharic MLHFQ scores and its subscales showed a moderate negative correlation with social support, a related but distinct construct, supporting divergent validity (Table 8).

**Table 8:** Convergent and discriminant validity of the Amharic MLHFQ scores (*n* = 383), 2023

Measure	MLHFQ Total Score (r, *p*-value)	MLHFQ Physical Score (r, *p*-value)	MLHFQ Emotional Score (r, *p*-value)
PHQ-9 (Depression)	r = 0.77., *p* < 0.001	r = 0.67., *p* < 0.001	r = 0.67., *p* < 0.001
OSS (Social support)	r = −0.58, *p* < 0.001	r = −0.47, *p* < 0.001	r = −0.47, *p* < 0.001

### Known-groups validity of the MLHFQ

As hypothesised, participants who were older, unemployed, had lower educational levels, or had poor social support tended to have significantly different MLHFQ scores compared with their counterparts. For example, participants who were older (≥70 years old) had significantly higher MLHFQ scores compared to those aged 18–39 (*p* = 0.001), with a moderate effect size (η^2^ = 0.09). Similarly, participants with depression had significantly higher MLHFQ scores compared to those without depression (*p* = 0.001), with a moderate effect size (d = 0.50). Group comparisons of the mean MLHFQ scores across sociodemographic and clinical variables are presented in Table 9.

**Table 9:** Known-group validity analysis of the Amharic MLHFQ scores (*n* = 383), 2023

Variable	Group	Mean (SD)	*p*-value	Effect size (Cohen’s d/η^2^)
Age in years	18–39 40–69 ≥70	39.59 (19.59) 46.97 (17.55) 57.90 (21.20)	0.001	η^2^ = 0.09
Sex	Male Female	46.64 (20.35) 49.32 (19.09)	0.185	d = 0.14
Employment status	Employed Unemployed	42.62 (1.46) 53.04 (1.29)	0.001	d = 0.55
Educational level	No formal education Primary Secondary College and above	50.79 (19.13) 51.52(19.33) 44.62(20.50) 42.69(18.85)	0.003	η^2^ = 0.04
Social support	Poor Moderate Strong	59.66(17.51) 43.26(18.04) 35.38(14.21)	0.001	η^2^ = 0.27
NYHA class	Class I Class II Class III Class IV	41.05(18.69) 44.77(18.82) 54.11(16.97) 67.97(14.65)	0.001	η^2^ = 0.18
Medications taken daily	<5 ≥5	46.38(1.17) 53.30 (1.92)	0.008	d = 0.30
Comorbidities				
Hypertension	No Yes	45.85 (1.40) 50.79 (1.43)	0.015	d = 0.25
Diabetes mellitus	No Yes	43.58 (1.04) 64.90 (1.79)	0.001	d = 1.20
HIV/AIDS	No Yes	47.51(1.03) 58.05 (4.27)	0.023	d = 0.54
Renal disease	No Yes	47.92 (1.06) 49.31 (3.14)	0.717	d = 0.07
Cancer	No Yes	47.97 (1.01) 54.66 (4.33)	0.559	d = 0.34
General health perception	Excellent Very good Fair Poor	34.28(22.45) 39.81(17.41) 50.27(19.01 57.48(16.33)	0.001	η^2^ = 0.15
Depression	No Yes	32.37(12.96) 60.01(15.08)	0.001	d = 0.50

## Discussion

Heart failure (HF), one of the most common reasons for hospitalisation in Ethiopia, is associated with poor health outcomes, including lower HRQoL and significant mortality [31, 45, 46]. Assessing HRQoL is one way of evaluating the effectiveness of HF treatment [47]. MLHFQ is the most widely used instrument for assessing the HRQoL of people with HF. This study aimed to translate culturally adapt and validate the Amharic version of the MLHFQ for use among people with HF in Ethiopia. To the best of our knowledge, this is the first study evaluating the psychometric properties of the MLHFQ in this population. This study involves a rigorous translation and cultural adaptation process, followed by assessment of reliability and evaluation of validity.

The translation and cultural adaptation of the MLHFQ for use in Ethiopia followed a rigorous and systematic process to ensure its relevance and clarity within the local context. This process adhered to standard translation guidelines [34], including forward and backward translation, to preserve the accuracy of the original content while adapting language and expressions to reflect cultural nuances. The adaptation phase also involved pre-testing the translated version with a small sample of people with HF to assess clarity, cultural appropriateness, and relevance of the items. Feedback from participants led to minor revisions to improve comprehension. Face and content validity were confirmed through expert review, ensuring that items were culturally relevant, clear, and representative of the construct. This process ensured that the adapted version of the MLHFQ is a linguistically and culturally appropriate tool for assessing the HRQoL of Ethiopian people with HF, thereby contributing to the growing body of research conducted in diverse cultural settings.

A reliability test using Cronbach’s alpha was performed to assess the consistency and accuracy of the Amharic MLHFQ. The reliability test results indicated that the overall MLHFQ showed acceptable internal consistency, with a Cronbach’s α coefficient of 0.90 for the overall MLHFQ and 0.84–0.87 for the physical and emotional subscales. This was consistent with the original model [36] and other two-factor model validation studies [21, 48]. The inter-item correlations were also acceptable, suggesting that each item contributes meaningfully to the overall scale without redundancy. In addition, the split-half reliability results confirmed the stability of the scale, demonstrating that the two halves of the instrument yield consistent scores. These findings collectively suggest that the Amharic MLHFQ and its subscales exhibit sufficient internal consistency among the items within the tool for measuring the impact of HF on HRQoL.

While recent studies have proposed a three-factor model for the MLHFQ [26, 49, 50], our data demonstrated a better fit with the original two-factor model, which comprises physical and emotional domains. Consistent with findings from previous validation studies conducted in diverse populations [36, 51, 52], the model fit indices in this study revealed an acceptable overall fit of the data to the model, although a few items showed relatively low factor loadings. Factor loadings greater than ±0.33 are generally considered to meet the minimal level of practical significance [39, 44]. On this basis, the results showed that the original two-factor structure MLHFQ appears to be an appropriate and valid tool for measuring the HRQoL of people with HF in Ethiopia. Moreover, the strong correlation observed between depression and HRQoL provides evidence of convergent validity of the translated MLHFQ, supporting the construct validity of the tool. This validates the use of the Amharic MLHFQ in this population and suggest that the physical and emotional domains effectively summarise the significant impact of heart failure on HRQoL in the Ethiopian context. In addition, known-groups validity was demonstrated, as participants with a worse clinical status (higher NYHA class, on polypharmacy, presence of comorbidities, and depression) had significantly higher scores than those with a milder status. For example, patients with advanced heart failure and those with depression exhibited significantly higher MLHFQ scores, as hypothesised. These findings are consistent with previous study [29], indicating that the instrument is sensitive to meaningful clinical differences between groups.

These steps provide preliminary evidence for the reliability and validity of the Amharic version of MLHFQ in the Ethiopian context; however, it is important to recognize that validation is an ongoing process. While the results of our psychometric analysis showed an acceptable but marginal model fit, they indicate areas for improvement. Factors such as item-specific issues or sample characteristics may have influenced the model fit. These borderline findings highlight the need for future research with larger and more diverse samples, which may strengthen the psychometric properties of the tool and improve model fit. The reported fit indices for the two-factor CFA were estimated based on a model with multiple specified error covariances. While this approach improved model fit, it may limit the generalizability and interpretability of the factor structure. Refining the factor structure, including exploring the potential existence of a third factor, may offer deeper insights into the multidimensional impact of heart failure on HRQoL. Such efforts are essential to ensure that the MLHFQ continue to consistently and accurately measure HRQoL as intended, while accounting for the unique cultural context, language nuances, and social norms of the Ethiopian population.

## Conclusion

The Amharic version of the MLHFQ demonstrated acceptable psychometric properties. The internal consistency test results for the overall MLHFQ and its subscales were acceptable, and the construct validity analyses provided adequate evidence supporting the validity of the tool. These findings suggest that the Amharic MLHFQ is a reliable, culturally adapted, and valid instrument for measuring HRQoL in people with HF in Ethiopia, supporting its application in both research and clinical setting. Future research is recommended to strengthen and confirm these findings in larger and diverse population.

## Data Availability

All data analysed in this study are available from the corresponding author upon a reasonable request.

## Abbreviations

AIDS: Acquired Immunodeficiency Syndrome
CFA: Confirmatory Factor Analysis
COPD: Chronic Obstructive Pulmonary Disease
CVI: Content Validity Index
HF: Heart Failure
HRQoL: Health-Related Quality of Life
MLHFQ: Minnesota Living with Heart Failure Questionnaire
NYHA: New York Heart Association
PHQ-9: Patient Health Questionnaire
OSSS-3: Oslo Social Support Scale
SD: Standard Deviation
